# The Clinical and Economic Impact of Inaccurate EGFR Mutation Tests in the Treatment of Metastatic Non-Small Cell Lung Cancer

**DOI:** 10.3390/jpm7030005

**Published:** 2017-06-28

**Authors:** Mindy M. Cheng, John F. Palma, Sidney Scudder, Nick Poulios, Oliver Liesenfeld

**Affiliations:** 1 Roche Molecular Systems, Inc., 4300 Hacienda Dr., Pleasanton, CA 94588, USA; sid.scudder@roche.com (S.S.); nick.poulios@roche.com (N.P.); oliver.liesenfeld@roche.com (O.L.); 2 Roche Sequencing Solutions, 4300 Hacienda Dr., Pleasanton, CA 94588, USA; john.palma@roche.com

**Keywords:** epidermal growth factor receptor (EGFR), non-small cell lung cancer (NSCLC), molecular diagnostic assay, companion diagnostic test, in vitro diagnostic (IVD), health care costs

## Abstract

Advances in personalized medicine are supported by companion diagnostic molecular tests. Testing accuracy is critical for selecting patients for optimal therapy and reducing treatment-related toxicity. We assessed the clinical and economic impact of inaccurate test results between laboratory developed tests (LDTs) and a US Food and Drug Administration (FDA)-approved test for detection of epidermal growth factor receptor (EGFR) mutations. Using a hypothetical US cohort of newly diagnosed metastatic non-small cell lung cancer (NSCLC) patients and EURTAC (erlotinib versus standard chemotherapy as first-line treatment for European patients with advanced EGFR mutation-positive non-small-cell lung cancer) clinical trial data, we developed a decision analytic model to estimate the probability of misclassification with LDTs compared to a FDA-approved test. We estimated the clinical and economic impact of inaccurate test results by quantifying progression-free and quality-adjusted progression-free life years (PFLYs, QAPFLYs) lost, and costs due to incorrect treatment. The base-case analysis estimated 2.3% (*n* = 1422) of 60,502 newly diagnosed metastatic NSCLC patients would be misclassified with LDTs compared to 1% (*n* = 577) with a FDA-approved test. An average of 477 and 194 PFLYs were lost among the misclassified patients tested with LDTs compared to the FDA-approved test, respectively. Aggregate treatment costs for patients tested with LDTs were approximately $7.3 million more than with the FDA-approved test, due to higher drug and adverse event costs among patients incorrectly treated with targeted therapy or chemotherapy, respectively. Invalid tests contributed to greater probability of patient misclassification and incorrect therapy. In conclusion, risks associated with inaccurate EGFR mutation tests pose marked clinical and economic consequences to society. Utilization of molecular diagnostic tests with demonstrated accuracy could help to maximize the potential of personalized medicine.

## 1. Introduction

At the core of personalized medicine is a belief that genome-based medicine will lead to greater efficiencies in healthcare via informed predictions about individuals’ susceptibility to disease, risk of progression, and treatment outcomes. An underlying assumption associated with this belief is that the molecular diagnostic tests used to analyze cellular biomarkers or genetic alterations are clinically validated, precise, and provide reliable information to healthcare providers, enabling them to correctly assess risk and make better-informed treatment decisions. The current global regulatory framework for molecular diagnostic tests, including companion diagnostics, is fragmented and inconsistent. Challenges still exist toward ensuring the quality, safety, and effectiveness of molecular diagnostic tests, due to lack of uniform evidence requirements by the various regulatory entities that oversee the development and provision of diagnostic tests, and the clinical laboratories in which the tests are performed [1,2]. Additionally, there is no standard health technology assessment (HTA) process for evaluating the value of molecular diagnostics, and there is a lack of guidance on how to measure the benefits of molecular diagnostic tests, appropriate study design, or test performance requirements [3]. While it is well-understood that molecular diagnostics are a critical component to personalized medicine, the test performance and value of many of the tests routinely used to inform patient care is uncertain.

In the US, one way that in vitro diagnostic tests (IVDs), including molecular diagnostics, may be commercialized for clinical use, is upon approval or clearance by the US Food and Drug Administration (FDA) per the Medical Device Amendments to the Federal Food, Drug, and Cosmetic Act (FD&C Act) [4]. As part of a premarket approval application, manufacturers are required to conduct rigorous technical performance validation studies (e.g., accuracy, reproducibility, reliability, sensitivity, specificity, limit of detection, inhibition, inclusivity, stability, etc.) to robustly demonstrate a test’s analytical validity (how well a test detects the presence of the intended analyte) and clinical validity (how well the presence or absence of the intended analyte predicts a clinical condition or predisposition in a patient) [2,4]. Separately, hospitals, universities, and commercial laboratories may use their own components and procedures to develop diagnostic tests for commercial use within a single laboratory facility irrespective of whether a FDA-approved IVD is available for the same purpose; these are referred to as laboratory developed tests (LDTs) [4]. Laboratories that develop their own tests used for clinical testing of patient specimens are regulated by the Clinical Laboratory Improvement Amendments (CLIA) program, primarily overseen by the Centers for Medicare and Medicaid Services (CMS). The CLIA program seeks to ensure the quality of laboratory facilities through focusing on quality control of testing procedures and appropriate training of laboratory personnel. Unlike FDA requirements, the CLIA program does not necessarily require demonstration of a test’s analytical and clinical validity, which often involves complex and multi-site trial designs [2,4]. Compliance with CLIA regulations may attest to quality standards of the laboratory facility and personnel, but does not ensure that LDTs are accurate and reliable in aiding clinical decision-making. There is no systematic assessment process in the US for LDT accuracy and test performance (sensitivity and specificity). As such, there is limited evidence available in the public domain regarding the performance of most LDTs routinely used to diagnose disease or aid in clinical decision-making [2,4].

Along with the proliferation of many new targeted cancer therapies, there has also been a proliferation in the number of highly complex molecular diagnostic tests that detect clinically relevant tumor biomarkers and aid in the identification of patients for targeted therapy [5]. For example, activating mutations in the tyrosine kinase domain of the epidermal growth factor receptor (EGFR) have been identified as an oncogenic driver in non-small cell lung cancer (NSCLC) cases [6]. First and second generation anti-EGFR tyrosine kinase inhibitors (TKIs) (e.g., erlotinib, gefitinib, afatinib) are first-line therapies for patients with EGFR mutation positive NSCLC, while conventional chemotherapy is recommended for patients who are EGFR wild type [7]. International treatment guidelines call for molecular diagnostic testing for the detection of EGFR-sensitizing mutations, as an aid to treatment selection for NSCLC patients with non-squamous histology [7,8,9]. LDTs for EGFR mutation testing are common and may be developed using polymerase chain reaction (PCR) or sequencing techniques. Very little information is available regarding the test performance of these LDTs, and there are no clinical guidelines about which testing platform or method offers optimal results [10]. Given the importance of EGFR mutation testing for therapy selection and the differential safety and effectiveness of TKI therapies compared to conventional chemotherapy for the treatment of metastatic NSCLC, there are significant clinical and economic consequences for incorrect (false positive (FP) and false negative (FN)) molecular diagnostic test results. In the case of EGFR mutation status misclassification, the consequence of FN results is greatest when patients with EGFR mutations are incorrectly classified as wild type and treated with chemotherapy, denying them the survival benefits associated with TKI therapy. In addition to erroneous results, invalid or delayed results due to technical errors and/or the presence of inhibitors also pose a challenge for the laboratory (need to re-run samples), and to patients (delay in initiation of appropriate therapy, or an additional biopsy if no residual sample is available).

Although diagnostic errors are common across healthcare settings, the topic has only recently received more attention due to a series of notable public health incidents where inaccurate diagnostic test results have caused harm to patients [1,4,11,12]. In 2014, the FDA stated intent to issue a new regulatory oversight framework for higher-risk LDTs, including companion diagnostics. However, the final guidance has not yet been released; it is uncertain if and when FDA will release the final guidance on this topic. As a result, inconsistencies in regulatory oversight and uncertain molecular diagnostic test performance remain open policy issues. The objective of this study was to use available data from the published literature in a case study to assess potential clinical and economic consequences of inaccurate EGFR mutation test results with LDTs compared to a FDA-approved IVD among a hypothetical cohort of newly diagnosed metastatic NSCLC patients in the US.

## 2. Materials and Methods

### 2.1. Study Design

We developed a decision analytic model to estimate the probability of test misclassification (FP and FN) by the cobas EGFR Mutation Test (Roche Molecular Systems, Pleasanton, CA, USA) or a combination of LDTs developed by the Laboratory of Oncology at the Germans Trias i Pujol Hospital (Barcelona, Spain) that were used to screen patients in the “erlotinib versus standard chemotherapy as first-line treatment for European patients with advanced EGFR mutation-positive non-small-cell lung cancer” (EURTAC) clinical study, a randomized phase III trial that assessed the safety and efficacy of erlotinib compared with standard chemotherapy as a first-line treatment for advanced EGFR mutation positive NSCLC [13,14]. We applied epidemiological estimates to the 2015 incidence of lung cancer cases to isolate a hypothetical US cohort of newly diagnosed patients with NSCLC tested for EGFR mutation. Next, we used the decision analytic model to estimate the probability of test misclassification among the hypothetical patient cohort and projected the clinical impact of FP and FN diagnostic test results by quantifying the average progression-free life years (PFLYs) and quality-adjusted progression-free life years (QAPFLYs) lost due to test misclassification and incorrect treatment selection. We estimated the subsequent cost impact of FP and FN test results to a healthcare payer, primarily US Medicare, by summing the cost of therapy and the cost to treat grade 3–4 adverse events among misclassified patients. In addition to inaccurate test results, we also determined the proportion of invalid results generated by LDTs compared to the cobas test.

### 2.2. Patient Population

The patient cohort was based on the 2015 projected incidence of lung cancer in the US, to which epidemiological estimates were applied to approximate the number of patients diagnosed with metastatic NSCLC (adenocarcinoma, large cell, or unspecified histology), assumed eligible for tissue biopsy and tested for EGFR mutation status. The US population prevalence of metastatic EGFR mutation positive NSCLC tumors was assumed to be the underlying true classification status of the patients. Table 1 describes the population and epidemiological estimates used to derive the national analytic patient cohort.

### 2.3. Decision Analytic Model

**Figure 1:**
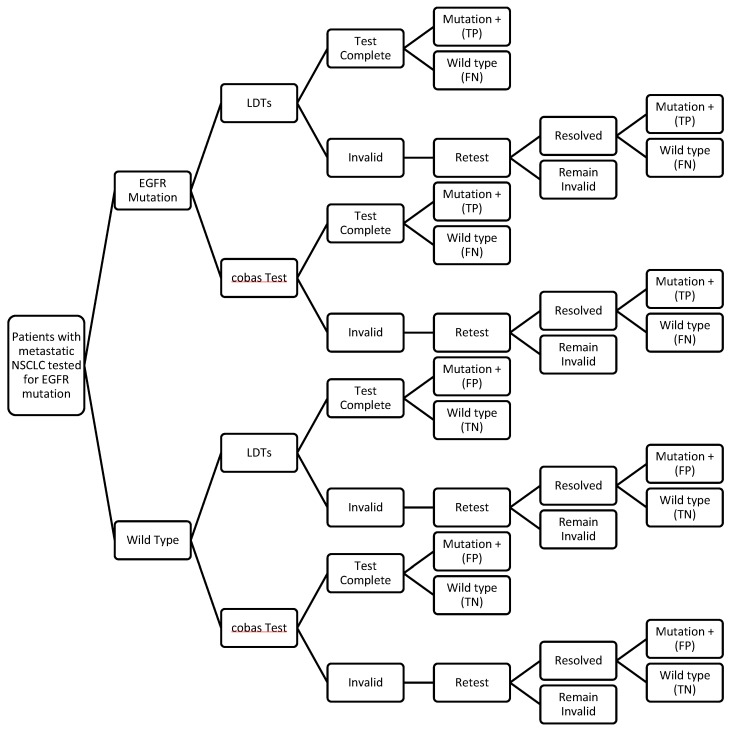
Decision Analytic Model. LDTs: Laboratory developed tests. EGFR: Epidermal growth factor receptor.

In the decision analytic model (Figure 1), all samples regardless of underlying mutation status could be tested with either the FDA-approved cobas test or with LDTs. With each testing platform, a proportion of tests were assumed to yield invalid results due to varying reasons. These samples were assumed to be re-tested once using the same test platform. Patients with EGFR mutation positive tumor samples were assumed to be treated with erlotinib while patients with wild type tumor samples were assumed to be treated with carboplatin and pemetrexed, a common chemotherapy regimen in the US for treating metastatic NSCLC [16]. Patients with samples that remained invalid after re-test and undetermined EGFR mutation status were assumed to be treated with chemotherapy.

### 2.4. Test Performance

The test performance estimates used in the base-case analysis were derived from a previously published clinical validation study conducted on specimens from the EURTAC clinical trial in which the cobas EGFR Mutation Test results were retrospectively compared to the original LDT results of specimens from patients screened for the trial. The clinical validation study design included a direct comparison of test performance between the cobas test and the LDTs, which provided a unique dataset that enabled the base-case analysis. The clinical validation study used massively parallel pyrosequencing (MPP) to resolve discrepant results between the cobas test and the LDTs [13]. The sensitivity and specificity of each testing platform were calculated, assuming that concordant test results between the cobas test and LDTs were “true” and that MPP revealed the “true” classification status among discordant test results due to a more sensitive testing methodology. The estimates of invalid test results for each platform were obtained from the same study. Table 2 presents the test performance data derived from the clinical validation study and used in the base-case analysis.

### 2.5. Clinical Inputs

The safety and efficacy of erlotinib among EGFR mutation positive patients was informed by the EURTAC clinical trial [14]. Progression-free survival (PFS) was used in this analysis as the measure of treatment benefit; overall survival (OS) between treatment arms could not be evaluated because 76% of patients in the standard chemotherapy group crossed over to erlotinib at progression. The safety and efficacy of the chemotherapy regimen was informed by a phase II randomized clinical trial that evaluated carboplatin and pemetrexed as first-line treatment in chemo-naïve patients with locally advanced or metastatic NSCLC [18]. The efficacy of erlotinib among EGFR wild type patients was informed by a phase III trial that compared gefitinib with a regimen of carboplatin and paclitaxel for treatment of advanced NSCLC [19]. It was assumed that gefitinib efficacy among EGFR wild type patients would be similar to erlotinib and would serve as an appropriate proxy due to lack of alternative published PFS Kaplan-Meier curves for erlotinib among EGFR wild type patients. We assumed that EGFR wild type patients treated with an EGFR TKI would have the same risk of treatment-related grade 3–4 adverse events as EGFR mutation positive patients. The areas under the published PFS Kaplan-Meier curves (without extrapolation) presented in all trials were used to estimate the mean duration of PFS benefit for each treatment regimen. The clinical estimates used in the base-case analysis are described in Table 3. We obtained utility estimates associated with each adverse event from the published literature and estimated mean QAPFLYs for the treatment cohorts based on the proportion of patients who experienced grade 3–4 adverse events as reported in the respective trials. Table 4 presents the utility estimates used to calculate QAPFLYs for the patient cohorts.

### 2.6. Cost Inputs

The down-stream cost impact of FP and FN results were estimated by summing the total costs of treatment (drugs, drug administration, and prophylactic medications) and the cost of treating grade 3–4 adverse events. Total drug costs were calculated by multiplying drug unit costs by the total administered dose over the median duration of treatment reported in the clinical trials. The carboplatin dose (AUC 6) was calculated using the Calvert formula and based on an average 64-year old male with mean weight of 82.05 kg and serum creatinine of 1 mg/dL [25]. For drugs with dosages based on body surface area (BSA), we assumed a mean BSA of 1.79 m^2^ from a study of adult cancer patients in the United Kingdom [26]. We used CMS July 2015 average sales price (ASP) for Medicare part B drugs to calculate costs for the chemotherapy regimen, including pre-medications, and the 2015 wholesale acquisition cost (WAC) for erlotinib [27]. We referred to a Medicare billing and coding guide to calculate drug administration costs for each regimen using national rate estimates [28]. Table 5 shows the inputs used to calculate total drug and drug administration costs per patient.

We estimated the cost of treating each adverse event by first assuming a typical care setting based on clinical experience. For adverse events which were assumed to be treated in a hospital inpatient setting, we applied recent estimates of national average hospital costs from the Agency for Healthcare Research and Quality’s Healthcare Cost and Utilization Project (AHRQ HCUPnet) database using primary International Classification of Diseases, ninth edition (ICD-9) diagnosis codes, and inflated the costs to 2015 values using the consumer price index for medical care [29]. For adverse events generally treated in an outpatient setting, we assumed primary treatment procedures based on clinical experience, obtained procedural reimbursement costs from the recent CMS proposed rule for Medicare hospital outpatient payments, and calculated therapy costs using the CMS July 2015 average sales price (ASP) for Medicare part B drugs [27,30]. The estimated costs to treat therapy-related adverse events are described in Table 6. Discounting was not applied to costs or outcomes in this analysis due to the relatively short time frame of projected survival for the patient cohort.

In the US and other countries, the cost to payers for EGFR mutation testing is analyte-specific and generally not differentiated between LDTs and regulatory-approved test kits; laboratories are reimbursed the same rate for EGFR mutation testing regardless if they used their own “home-brew” assay or a commercial, regulatory-approved test. There is one exception in the US with the MolDx Program administered by Medicare contractors that allows for a higher reimbursement rate to registered labs if EGFR mutation testing is performed with a FDA-approved test kit relative to LDTs. However, given that the objective of this study was to assess the down-stream cost consequences associated with incorrect test results, and was not intended as a cost-effectiveness analysis of EGFR mutation testing strategy, the cost to payers for diagnostic testing was not included in the analysis.

### 2.7. Scenario Analyses

The test performance data used in the base-case analysis were derived from a clinical validation study of the cobas test in which specimens from patients enrolled in the EURTAC clinical trial were prospectively screened using LDTs and retrospectively tested with the cobas test [13]. Due to lack of alternative data sources regarding the general test performance of LDTs for EGFR mutation testing, it is uncertain how well the LDTs developed specifically for research and used in a controlled clinical trial setting reflected the real-world test performance of LDTs routinely developed by different laboratories [4,31]. Given the uncertainty with the LDT estimates used in the base-case analysis, we conducted scenario analyses to explore the impact of varying LDT test performance on the results. We used a Diagnostic Assessment conducted by the National Institute for Health and Care Excellence (NICE) in the United Kingdom to inform the LDT test performance estimates explored in scenario analyses [32]. We also explored the impact of higher invalid rates by analyzing a scenario assuming that 20% of LDT test results were invalid while holding other base-case estimates the same. Table 7 summarizes the test performance inputs used in the base-case analysis and each of three scenario analyses.

## 3. Results

**Figure 2:**
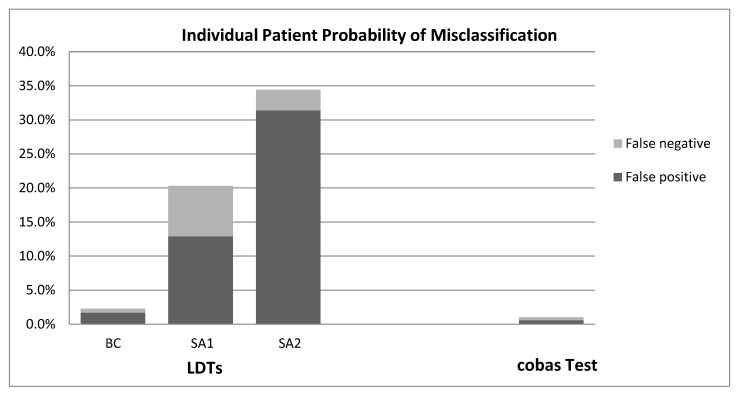
Individual Patient Probability of Misclassification by LDTs and the cobas Test. LDTs = Laboratory-developed tests; BC = Base-case; SA = Scenario analysis.

Using the referenced data inputs, it was estimated that 2.4% (*n* = 1051 FP, 371 FN) of 60,502 patients in the US with newly diagnosed metastatic NSCLC and tested for EGFR mutation would be misclassified if all patients were tested with LDTs compared to 1% (*n* = 353 FP, 224 FN) of patients if the same cohort was tested using the FDA-approved cobas EGFR Mutation Test. Figure 2 shows the individual patient probability of FP or FN test results from LDTs relative to the cobas test.

Additionally, it was estimated that 0.6% (*n* = 378) of the patient cohort tested with LDTs would have unresolved invalid tests and would be assumed as treated with chemotherapy. Among these patients, it was projected that 72 would actually have an EGFR mutation and therefore be incorrectly treated.

As a result of inaccurate and invalid diagnostic test results and subsequently being treated with an “incorrect” treatment regimen, it was estimated that if the entire patient cohort was tested with LDTs, it would lose at least an average of 477 progression-free life years (PFLYs) compared to 194 PFLYs if the cohort was tested with the FDA-approved test. This translated into approximately four months of lost PFS per any misclassified patient. When the survival was quality-adjusted to account for the impact of treatment-related severe adverse events, it was projected that the cohort tested with LDTs would lose at least an average of 319 quality-adjusted progression-free life years (QAPFLYs) (approximately five months of quality-adjusted PFS per misclassified patient) compared to 131 QAPFLYs (approximately three months of quality-adjusted PFS per misclassified patient) with the FDA-approved cobas test.

**Figure 3:**
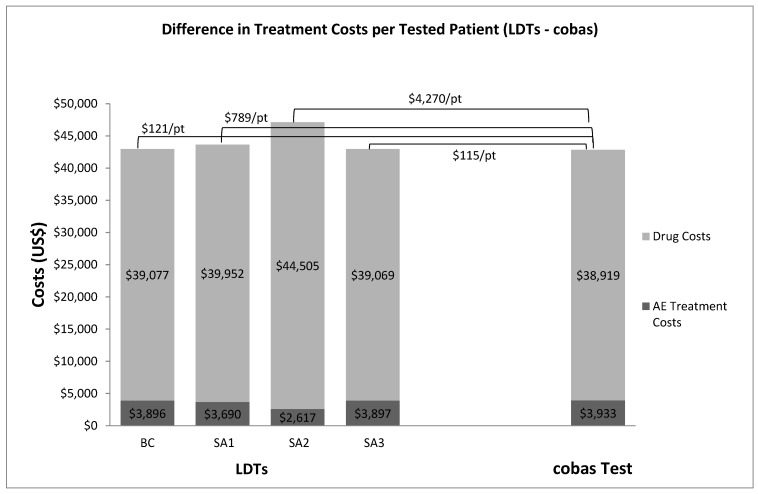
Difference in treatment costs per tested patient between LDTs and the cobas Test.

If the national analytic cohort of 60,502 patients was tested for an EGFR mutation with LDTs, the total aggregate treatment cost (drugs, drug administration, adverse events) to Medicare was estimated at $2,599,931,837 compared to $2,592,625,528 if the cohort was tested with the FDA-approved test. The difference of approximately $7.3 million in aggregate treatment costs between testing with LDTs and the FDA-approved cobas test was driven by higher drug costs among patients who tested FP and were incorrectly treated with EGFR TKI therapy, as well as higher costs to treat adverse events among patients who tested FN and were incorrectly treated with chemotherapy. Approximately 3% and 1% of the total aggregate treatment cost associated with LDTs and the FDA-approved cobas test, respectively, was attributed to misclassified patients. Figure 3 shows the difference in treatment costs per tested patient with LDTs compared with the cobas test in the base-case and scenario analyses.

The scenario analyses show that if the average test performance of EGFR mutation LDTs were approximately 61% sensitive and 84% specific, an estimated 20% (*n* = 12,247) of the 2015 US patient cohort tested for EGFR mutations with LDTs were projected to be misclassified, 12.9% FP (*n* = 7792) and 7.4% FN (*n* = 4455). Consequent to the misclassification and incorrect treatment, an average of 4104 PFLYs or 2758 QAPFLYs would be lost among this patient cohort relative to all patients correctly classified; 23% (~$607 million) of total aggregate costs would be attributed to misclassified patients. Subsequently, if LDTs were 84% sensitive and 61% specific [32], an estimated 34.4% (*n* = 18,993 FP, 1828 FN) of the patient cohort would be incorrectly treated due to inaccurate test results, with a projected loss on average of 5848 PFLYs or 3839 QAPFLYs. It was estimated that 39% (~$1 billion) of aggregate treatment costs would be attributed to misclassified patients with a significant proportion attributed to higher drug costs for patients incorrectly treated with EGFR TKI therapy and higher costs to treat adverse events among patients incorrectly treated with chemotherapy. If LDTs had a higher invalid rate of up to 20% (sensitivity 98.1%, specificity 99.3% assumed in the base-case), it was estimated that 0.8% (*n* = 491) of the national analytic patient cohort would have an unresolved test and be treated with chemotherapy by default. If EGFR mutation prevalence is 19% [16], then it was estimated that 93 of these patients would be incorrectly treated.

## 4. Discussion

We developed a decision analytic model to evaluate the probability of diagnostic error with LDTs for EGFR mutation testing compared to a FDA-approved test (cobas EGFR Mutation Test). We applied the decision analytic model to estimate the clinical and economic consequences of inaccurate test results on a cohort of patients with newly diagnosed metastatic NSCLC in the US. The primary limitation of the analysis was the lack of published data regarding test performance and the accuracy of the numerous EGFR mutation LDTs available across various hospitals, laboratories, and medical centers. For the base-case analysis, we used the best available data from a clinical validation study of the cobas test in which the study design compared the cobas test results retrospectively to results from LDTs used in the EURTAC clinical trial. This validation study provided a unique dataset from a direct comparison of the cobas test and LDTs for EGFR mutation testing. We noted that the LDTs used in the EURTAC clinical trial had similar sensitivity and specificity to the cobas test with only a slightly higher invalid test rate (cobas test: sensitivity 98.1%, specificity 99.3%, invalid rate 8.9%; LDTs: sensitivity 96.8%; specificity 97.8%, invalid rate 15.6%); we used these estimates in the base-case analysis to understand the clinical and economic impact of even small differences in test performance.

With sparse evidence describing the overall test performance of LDTs, it is uncertain how well the LDTs used in the EURTAC clinical study reflect the quality and real-world test performance of the various LDTs used across different laboratories for EGFR mutation testing. In Europe, many countries have external quality assessment (EQA) programs that utilize an independent external agency to objectively check laboratory results and testing methods [33]. In one study that evaluated one hundred and seventeen labs across thirty European countries for EGFR mutation testing, only 72% of the laboratory participants passed the quality assessment, with false negative and false positive results being the main sources of error [33]. In another EQA conducted in the United Kingdom, 24% of labs had genotyping errors in the first run, 6.7% in the second run, and 6.4% in the third run. The assessment observed there was a range of testing methodologies applied across different labs and wide variation in the degree of interpretation provided on the test reports [34]. Given that the US does not have similar systematic quality assessment programs, we had very limited information about the robustness of laboratory methodologies and the quality of laboratory-developed “home-brew” tests. Given the uncertainty with LDT performance, we conducted scenario analysis to evaluate the impact if LDT performance varied. For the scenario analysis, we assumed LDT performance estimates derived from a Diagnostic Assessment conducted in the UK, which identified only six studies in the published literature that provided data on the accuracy of EGFR mutation testing for predicting response to TKI therapy [32].

The base-case analysis showed that even very low individual patient probabilities of inaccurate test results (FP or FN) led to clinical and economic consequences at the population level in terms of the aggregate impact of incorrect treatment, negative clinical outcomes, morbidity and pre-mature mortality. Invalid test results were also impactful, due to greater probability that the uncertainty led to patient misclassification and incorrect therapy.

The magnitude of impact of inaccurate testing estimated in this analysis is likely not generalizable across all molecular diagnostic tests and tumor types, as the clinical and cost consequences of patient misclassification largely depend on the differential safety and efficacy of the indicated treatment regimens and the size of the population afflicted. For certain assays, high sensitivity (minimize FN) will be more important than specificity toward ensuring appropriate treatment of patients, whereas in other cases, high specificity (minimize FP) is a priority, in order to minimize patient harm and achieve optimal treatment outcomes.

A limitation associated with using the PFS endpoint is that it fails to capture survival time post-disease progression, and it was therefore likely that this study underestimated the “true” burden of inaccurate EGFR mutation tests on society, as the analysis also did not capture indirect or opportunity costs, nor other quality of life impacts associated with diagnostic error, incorrect treatment or treatment uncertainty. Given the available data, this study provided a base-line estimate of the impact of inaccurate EGFR mutation testing and highlights the importance of a holistic total cost of care perspective. When laboratories make decisions about product adoption, a primary focus on utilizing test platforms with lower adoption costs favoring LDTs fails to take into consideration the potential down-stream costs to patients and the broader healthcare system if LDT performance is uncertain relative to clinically validated FDA-approved products. From a total cost of care perspective, cost-savings in the laboratory budget may translate into unnecessary spending (medical waste) elsewhere in the system (e.g., pharmacy or hospital budget). Toward this end of reducing the societal burden of inaccurate testing, priority should be placed on adopting diagnostic tests with robust evidence of clinical validity and demonstrated analytical accuracy and reliability. This study is not intended to suggest that LDTs are somehow “bad” and should be avoided, as we recognize that LDTs have a significant role in diagnostics and are important for many applications, such as rare disease testing or public health crises when regulatory-approved, commercial test kits are not available. The intent of this analysis was to highlight the differences between evidence-based requirements and test performance data between regulatory-approved tests and LDTs, and to use a case example of EGFR mutation testing to demonstrate the potential clinical and economic consequences of incorrect treatment decisions due to diagnostic tests with uncertain test performance.

Given the sparse level of evidence for many LDTs routinely used to guide clinical decision-making, the value of molecular diagnostic tests should not all be perceived as equal. FDA-approved IVDs with robust supporting evidence are differentiated due to a greater certainty in their ability to provide the correct results and thereby improve patient outcomes and healthcare efficiency. Vyberg and colleagues analyzed the socioeconomic consequences of inaccurate HER2 test results between regulatory-approved tests and LDTs for the treatment of breast cancer, and suggested that using regulatory-approved HER2 tests rather than LDTs could result in annual savings of $46 million, largely due to correct treatment with trastuzumab and avoiding treatment costs associated with disease recurrence and progression. Vyberg, et al. also suggested that for every $1 saved by laboratories using cheaper LDT reagents, the healthcare system is potentially burdened with approximately $6 in additional costs due to inaccurate testing and incorrect treatment [35]. Garrison and colleagues also examined the clinical and economic consequences of inaccurate HER2 testing on US patients with early-stage breast cancer and found that incorrect HER2 testing may contribute to total societal loss of up to $1 billion among a cohort of 12,025 misclassified patients [36]. In-line with our findings, Garrison, et al. demonstrated that the consequences of FP and FN test results differ such that FP results led to the use of HER2-targeted therapy for patients with little chance of benefit and yielded an increased risk of adverse events and higher treatment costs. Conversely, FN results denied patients potential quality of life and survival benefits associated with targeted therapy, and led to increased risk of disease recurrence and progression to metastatic breast cancer [36].

## 5. Conclusions

In order to realize the full potential of personalized medicine, these findings highlight how critical it is for laboratories to utilize companion diagnostic molecular tests with robust evidence of accuracy, test performance, and clinical validation that includes outcomes for the intended population. Diagnostic errors pose clinical and economic consequences to society and warrant consideration for consistent regulatory review and comprehensive quality assessment testing of all molecular diagnostic tests, regardless of developer, to better control the safety and effectiveness of diagnostic tests routinely used to inform patient care.

## Figures and Tables

**Table 1:** Epidemiological estimates used to calculate the national analytic patient cohort.

Parameter	Estimate	Reference
New lung cancer cases diagnosed in US in 2015	221,200	[15]
% Lung cancer cases that are NSCLC	85%	[16]
% NSCLC that are adenocarcinoma, large cell, or unspecified histology	73%	[16]
% NSCLC advanced or metastatic at diagnosis (Stage IIIb or IV)	58%	[16]
Diagnosed Cohort	79,608	
% Diagnosed patients tested for EGFR mutation in US	76%	[17]
National Analytic Cohort	60,502	
Prevalence of EGFR mutation in US	19%	[16]

NSCLC = Non-small cell lung cancer; EGFR = Epidermal growth factor receptor.

**Table 2:** Base-case estimates of test performance data after MPP resolution [13].

**Cobas Test Results**	**Mutation Status Based on MPP Resolution**		
	**Mutation +**	**Wild Type**	**Total**
Mutation +	151	2	153
Wild Type	3	276	279
**Total**	**154**	**278**	**432**
Sensitivity: 98.1% Specificity: 99.3% Invalid rate: 8.9%			
**LDT Results**	**Mutation Status based on MPP Resolution**		
	**Mutation +**	**Wild Type**	**Total**
Mutation +	149	6	155
Wild Type	5	272	277
**Total**	**154**	**278**	**432**
Sensitivity: 96.8% Specificity: 97.8% Invalid rate: 15.6%			

MPP = Massively parallel pyrosequencing; LDT = Laboratory developed test.

**Table 3:** Clinical estimates associated with erlotinib and chemotherapy treatment.

Survival	Chemotherapy (Carboplatin + Pemetrexed) [18]	Erlotinib (EGFR Mutation Positive) [14]	Erlotinib (EGFR Wild Type) [14]
Mean duration of PFS (months)	8.55	11.50	2.98 [19]
Grade 3–4 Adverse Events			
Anemia	7.7%		
Arthralgia		1.0%	1.0%
Diarrhea		5.0%	5.0%
Fatigue	7.7%	6.0%	6.0%
Febrile Neutropenia	5.1%		
Infection	2.6%		
Neuropathy		1.0%	1.0%
Neutropenia	25.6%		
Pneumonitis		1.0%	1.0%
Rash		13.0%	13.0%
Stomatitis	2.6%		
Thrombocytopenia	17.9%		

PFS = Progression-free survival; EGFR = Epidermal growth factor receptor.

**Table 4:** Utility estimates used to calculate quality-adjusted progression-free life years (QAPFLYs) associated with treatment.

Adverse Event	Utility Estimate	Reference
Base-line, stable disease, no toxicity	0.653	[20]
Anemia (grade 3–4)	0.583	[21]
Arthralgia (grade 3–4 )	0.589	[22]
Diarrhea (grade 3–4 )	0.606	[20]
Fatigue (grade 3–4 )	0.580	[20]
Febrile Neutropenia (grade 3–4 )	0.563	[20]
Infection (grade 3–4 )	0.423	[22]
Neuropathy (grade 3–4 )	0.620	[23]
Neutropenia (grade 3–4 )	0.563	[20]
Pneumonitis (grade 3–4 )	0.560	[24]
Rash (grade 3–4 )	0.621	[20]
Stomatitis (grade 3–4 )	0.610	[23]
Thrombocytopenia (grade 3–4 )	0.545	[22]

**Table 5:** Cost inputs used to calculate total treatment and treatment administration costs (2015$).

Drug	Administration Route	Unit Cost	Dose per Cycle	Drug Cost per Cycle	Drug Admin. Cost per Cycle	Duration of Treatment	Total Cost
Carboplatin	IV infusion	$3.85/50 mg	669.7 mg	$51.57	$136.55	6 cycles (median)	$1,129
Pemetrexed	IV infusion	$61.33/10 mg	895 mg	$5,489.04	$63.24	6 cycles (median)	$33,314
Folic acid *	Oral	Supplements not reimbursed by Medicare					Assume negligible
Vitamin B12 *	Subcutaneous injection	$4.39/1 mg			$53.52	3 total injections	$174
Dexamethasone *	Oral	$0.25/0.25 mg	24 mg	$24.00		6 cycles (median)	$144
Ondansetron *	Subcutaneous injection	$0.073/1 mg	24 mg	$1.75	$53.52	6 cycles (median)	$332
**Total Chemotherapy and Admin. Cost per Patient**							**$35,092**
**Total Erlotinib Cost per Patient**	**Oral**	**$223.63/150 mg**	**150 mg/day**			**246 days (median)**	**$55,013**

* Prophylactic/pre-medications for chemotherapy regimen. IV = Intravenous.

**Table 6:** Estimated costs to treat grade 3–4 adverse events.

Adverse Event	Setting of Care/Primary Treatment Procedure	Treatment Cost Estimate (2015$)	Reference
Anemia	Outpatient/Blood transfusion	$598	[30]
Arthralgia *			Clinical opinion
Diarrhea *			Clinical opinion
Fatigue *			Clinical opinion
Febrile Neutropenia	Inpatient	$20,254	[29]
Infection	Inpatient	$16,657	[29]
Neuropathy *			Clinical opinion
Neutropenia	Outpatient/Neupogen	$12,423	[27,30]
Pneumonitis	Inpatient	$14,097	[29]
Rash *			Clinical opinion
Thrombocytopenia	Outpatient/Blood transfusion	$795	[30]

* Assume minimal resources to treat symptoms; negligible costs.

**Table 7:** Summary of test performance inputs used in the base-case and scenario analyses.

	Sensitivity	Specificity	Invalid Rate	Reference
cobas test	98.1%	99.3%	8.9%	[13]
LDTs-Base-Case (“BC”)	96.8%	97.8%	15.6%	[13]
LDTs-Scenario 1 (“SA1”)	61.0%	84.0%	15.6%	[32]
LDTs-Scenario 2 (“SA2”)	84.0%	61.0%	15.6%	[32]
LDTs-Scenario 3 (“SA3”)	96.8%	97.8%	20.0%	Exploratory
